# Long-Term Effect of a Single Dose of Caffeine on Sleep, the Sleep EEG and Neuronal Activity in the Peduncular Part of the Lateral Hypothalamus under Constant Dark Conditions

**DOI:** 10.3390/clockssleep4020023

**Published:** 2022-05-25

**Authors:** Yumeng Wang, Tom Deboer

**Affiliations:** Department of Cell and Chemical Biology, Leiden University Medical Center, 2333 ZC Leiden, The Netherlands; y.wang.mcb@lumc.nl

**Keywords:** caffeine, electroencephalogram, rapid eye movement sleep, theta activity, constant darkness, lateral hypothalamus

## Abstract

Background: Caffeine is a central nervous system stimulant that influences both the sleep–wake cycle and the circadian clock and is known to influence neuronal activity in the lateral hypothalamus, an important area involved in sleep–wake regulation. Light is a strong zeitgeber and it is known to interact with the effect of caffeine on the sleep–wake cycle. We therefore wanted to investigate the long-term effects of a single dose of caffeine under constant dark conditions. Methods: We performed long-term (2 days) electroencephalogram (EEG)/electromyogram recordings combined with multi-unit neuronal activity recordings in the peduncular part of the lateral hypothalamus (PLH) under constant darkness in Brown Norway rats, and investigated the effect of a single caffeine treatment (15 mg/kg) or saline control given 1 h after the onset of the endogenous rest phase. Results: After a reduction in sleep and an increase in waking and activity in the first hours after administration, also on the second recording day after caffeine administration, rapid eye movement (REM) sleep was still reduced. Analysis of the EEG showed that power density in the theta range during waking and REM sleep was increased for at least two days. Neuronal activity in PLH was also increased for two days after the treatment, particularly during non-rapid eye movement sleep. Conclusion: Surprisingly, the data reveal long-term effects of a single dose of caffeine on vigilance states, EEG, and neuronal activity in the PLH. The absence of a light–dark cycle may have enabled the expression of these long-term changes. It therefore may be that caffeine, or its metabolites, have a stronger and longer lasting influence, particularly on the expression of REM sleep, than acknowledged until now.

## 1. Introduction

Caffeine is the most widely used psychoactive stimulant worldwide. It has numerous pharmacological and physiological effects, including increasing arousal, cardiovascular activity, respiration, renal activity, and alertness. In addition, it has effects on mood, memory, and cognitive performance [1,2,3]. Caffeine has a noticeable effect of increasing waking and locomotor activity, and decreasing the expression of slow waves in the non-rapid eye movement (NREM) sleep electroencephalogram (EEG) [1,4], which are thought to be markers of sleep homeostasis [5]. Caffeine works via antagonism of adenosine receptor signaling across almost all brain areas and generally induces an increase in neuronal activity [6]. The levels of adenosine in the brain increase during sleep deprivation and decrease during recovery [7,8]. Therefore, adenosine is thought to be one of the substances involved in the homeostatic regulation of sleep [9].

Most of the studies investigating the effect of caffeine on sleep analyzed only the first 24 h after acute administration of caffeine. However, caffeine may have a more prolonged effect on the sleep–wake cycle. The half-life of caffeine ranges from 0.7 to 1.2 h in rats after 5–10 mg/kg [10] and from to 2.5 to 4.5 h after 4 mg/kg in adult humans (280 mg/70 kg, 2–3 cups of coffee) [11]. Moreover, 15 mg/kg caffeine (corresponding to the ingestion of 300–400 mg of caffeine in a 70 kg adult human) [6] has an effect on the vigilance states, which seems to last for approximately 3 h in albino rats [1]. However, when closely investigating the metabolism of caffeine, the effect of caffeine may last longer. The pharmacokinetic profile of caffeine shows that it remains in the bloodstream approximately 350 min after oral administration of 5 mg/kg caffeine in mice [12]. After intravenous application of 15 mg/kg of caffeine, an increased concentration of caffeine was still detectable 10 h after administration [1,13]. Next to that, metabolites of caffeine, such as paraxanthine, are also known to function on adenosine receptors [14,15]. This suggests that caffeine and its metabolites may stay in circulation and influence sleep and waking for longer than previously reported.

Light is a strong zeitgeber that entrains the endogenous circadian rest activity rhythm to the external time [16,17,18]. Adenosine may also play a role in the functioning of the circadian clock. Application of an adenosine agonist attenuates light-induced phase shifts in behavior. This attenuation can be restored by an adenosine receptor antagonist [19,20]. It has been shown that caffeine lengthens the free-running period in mice under constant condition [21,22]. Interestingly, the effect of light on the circadian system is influenced by caffeine. It increased the light-induced phase-shift in mice and *Arvicanthis*, and enhanced the light-induced suprachiasmatic nucleus neural activity in mice [21,23,24,25]. Caffeine may, in that way, strengthen the rhythm of the endogenous circadian clock. These studies show that adenosine and caffeine not only affect sleep and waking, but also influence the period and light responsiveness of the circadian clock. Particularly, in a light–dark cycle, both the acute and the long-term effects on the second day may be influenced by the input of light on the circadian pacemaker. Nevertheless, there is virtually no study that investigates the sleep–wake cycle after caffeine administration under constant darkness.

Caffeine’s effect on arousal is central in origin. In rat studies, 10–30 mg/kg caffeine induced large levels of c-fos expression across the brain [26]. Although most of the expression was in the cortex, it was not limited to the cortex as it was also visible in the hypothalamus, including the orexin neurons in the peduncular part of the lateral hypothalamus (PLH) [27]. When and for how long this activation in the hypothalamus lasts is unclear. Most brain areas involved in sleep–wake regulation can be found in the hypothalamus and the pons [28,29]. They consist of two ascending pathways that promote waking and wake maintenance. The first releases acetylcholine, which is produced by neurons in the pedunculopontine and laterodorsal tegmental nucleus and is active during waking and REM sleep [30]. The second releases different monoamines from the locus coeruleus (noradrenaline), raphe (serotonin), ventral periaqueductal gray matter (dopamine), and tuberomammillary neurons (dopamine). These project to the lateral hypothalamus (LH), the basal forebrain, and the cerebral cortex [31] and are most active during waking, less active during NREM sleep, and silent during REM sleep. On the opposite side, the ventrolateral preoptic area (VLPO) is thought to mainly stimulate NREM sleep. The VLPO has outputs to the acetylcholine and monoamine nuclei involved in wake maintenance and is active during sleep, when it releases the inhibitory neurotransmitters galanin and GABA [32,33,34]. Together, this forms a self-reinforcing loop, which in electrical engineering is called a “flip-flop switch”, a term also coined for this system [28]. Orexin, released by specific neurons in the LH, is thought to stabilize this switch and to reinforce waking without inhibiting activity of the VLPO [28].

Based on the previous research summarized above, it is not unlikely that the effect of a single dose of caffeine is underestimated in situations where the effect of caffeine is tested under normal light–dark conditions. To further investigate these different aspects of the possible influence of caffeine, we assessed the effect of a single dose of 15 mg/kg caffeine on PLH neuronal activity, combined with vigilance state recordings in freely moving rats. Thus, in our experiment, we investigated the long-term (2 days) effect of a stimulatory concentration of caffeine on the sleep–wake cycle and EEG and locomotor activity under constant darkness. As light and caffeine interact in their effect on sleep and waking and the circadian clock, we decided to record in constant darkness to be able to distinguish the effect of a single dose of caffeine on both sleep–wake cycle and the circadian clock. In addition, to investigate the effect on neuronal activity in PLH, we recorded electrical multi-unit neuronal activity in this brain area.

## 2. Results

### 2.1. Effect of Caffeine on the Sleep–Wake Cycle and Locomotor Activity

**Figure 1:**
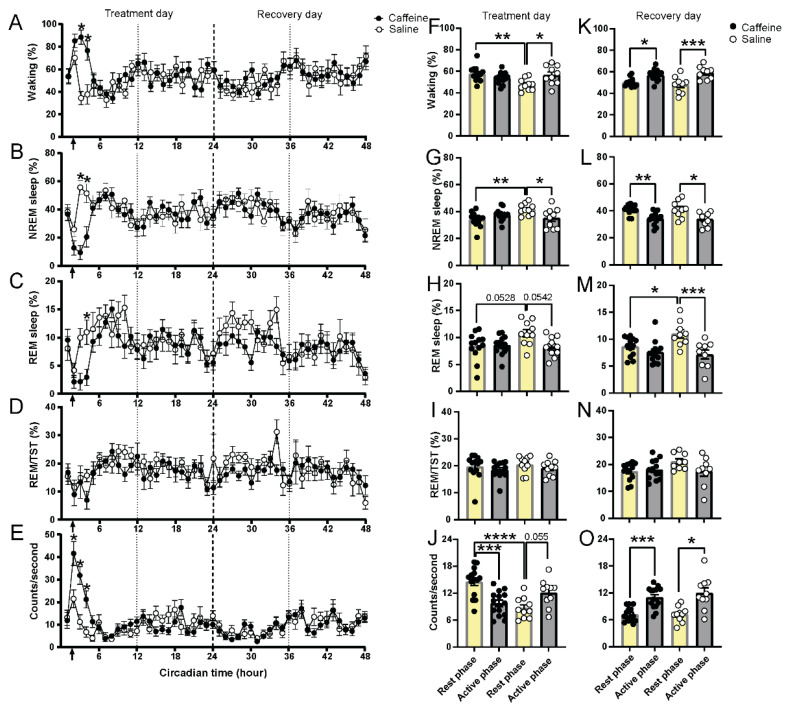
Vigilant states and locomotor activity of caffeine and saline treatment in 48 h recording under constant darkness. (**A**–**E**) Time course of waking, NREM sleep, REM sleep, REM sleep/total sleep time, and locomotor activity in 1 h value for caffeine (black, *n* = 13) and saline (white, *n* = 10) administration in 48 h. The first 24 h were considered as the treatment day; the second 24 h were considered as the recovery day. Arrows indicate the injection time (CT1-2). Asterisks indicate significant differences between caffeine and saline administration (* *p* = 0.05–0.0001, Bonferroni multiple comparisons test after significant two-way ANOVA, factors “treatment” or interaction of “circadian time” and “treatment”). (**F**–**J**) Rest and active phase values of waking, NREM sleep, REM sleep, REM sleep/total sleep time, and locomotor activity for caffeine (black, *n* = 13) and saline (white, *n* = 10) administration on treatment day. (**K**–**O**) Rest and active phase values of waking, NREM sleep, REM sleep, REM sleep/total sleep time, and locomotor activity for caffeine (black, *n* = 13) and saline (white, *n* = 10) administration on recovery day. CT0–CT12 was considered as the rest phase (yellow bar); CT12-CT24 was considered as active phase (gray bar). Asterisks indicate significant differences between caffeine and saline administration (* *p* < 0.05, ** *p* < 0.01, *** *p* < 0.001, **** *p* < 0.0001, Bonferroni multiple comparisons test after significant two-way ANOVA). Data are shown as mean ± SEM.

Immediately after the caffeine injection (15 mg/kg), waking and activity were increased and the amounts of NREM sleep and REM sleep were reduced (Figure 1). This initial effect lasted for approximately 2–3 h compared with saline injection (Figure 1A–E). After that, no differences were found in the hourly values of the vigilance states or activity level. Surprisingly, a single application of caffeine on the first day still influenced the occurrence of REM sleep on the second recovery day (Figure 1C). REM sleep was decreased by 21.5% during the subjective day (the rest phase of the animal where it normally is light) relative to saline on the second recovery day (Figure 1M). In contrast, no effects on the other vigilance states or activity levels were found on the second day (Figure 1F–O; Table 1). Moreover, after caffeine, the day–night differences in waking, NREM sleep, and REM sleep were diminished on the first day (Figure 1F–H). The day–night difference after caffeine treatment was restored on the second recovery day for waking and NREM sleep (Figure 1K,L), but not for REM sleep (Figure 1M).

The results obtained on the first day are in accordance with previous findings, where it was found that the effect of this dose of caffeine on waking, NREM sleep, and locomotor activity lasts for approximately 3 h following administration [1]. However, the influence on REM sleep time lasted considerably longer. Based on our findings, it seems that a single dose of 15 mg/kg caffeine can influence the occurrence of REM sleep for more than 24 h.

### 2.2. Effect of Caffeine on Vigilance State Episodes

**Figure 2:**
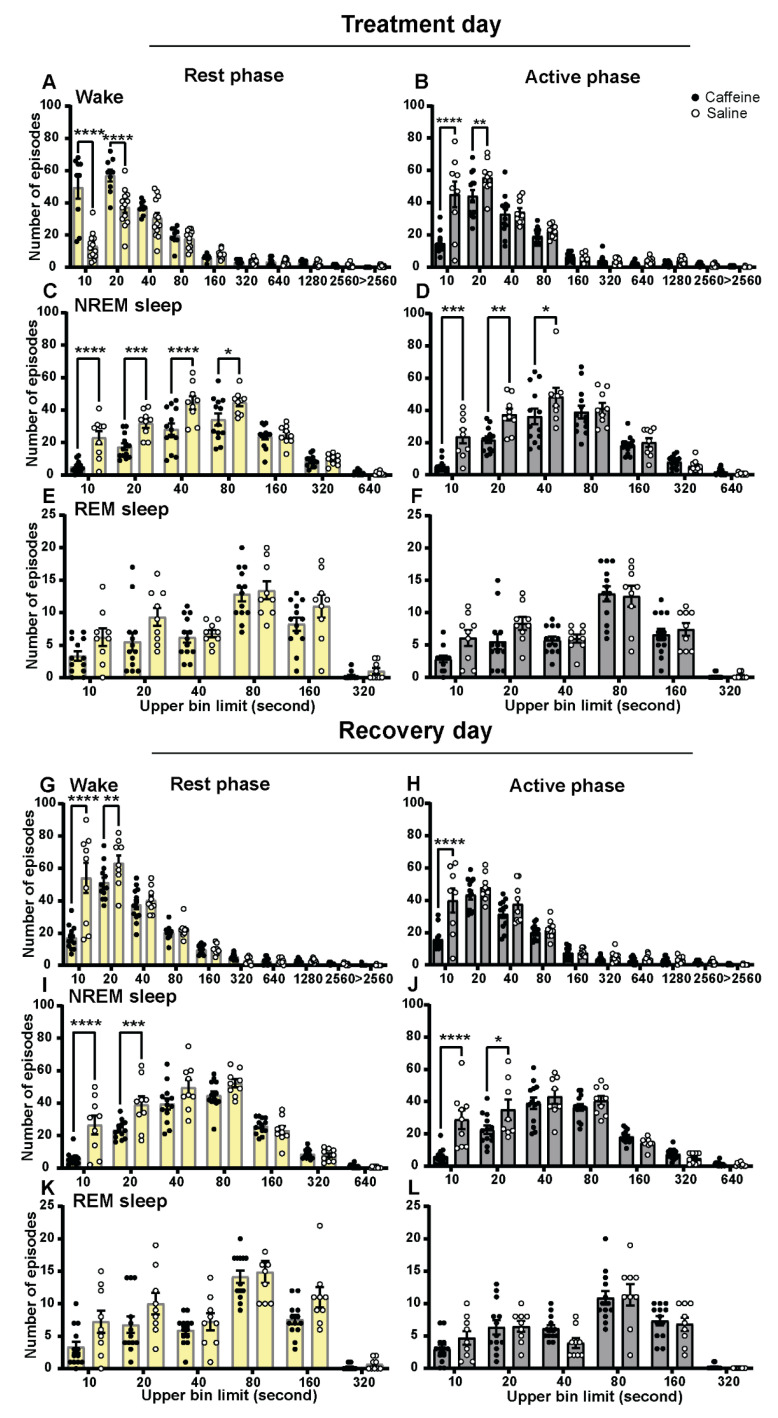
Episode duration histograms of rest phase and active phase. (**A**,**B**) Episode duration histograms of waking in the rest and active phase for caffeine (black, *n* = 13) and saline (white, *n* = 9) administration. (**C**,**D**) Episode duration histograms of NREM sleep in the rest phase and active phase for caffeine and saline. (**E**,**F**) Episode duration histograms of REM sleep in the rest phase and active phase for caffeine and saline. (**G**,**H**) Episode duration histograms of waking in the rest phase and active phase for caffeine (black, *n* = 13) and saline (white, *n* = 9) administration on recovery day. (**I**,**J**) Episode duration histograms of NREM sleep in the rest phase and active phase for caffeine and saline on recovery day. (**K**,**L**) Episode duration histograms of REM sleep in the rest phase and active phase for caffeine and saline on recovery day. * *p* < 0.05, ** *p* < 0.01, *** *p* < 0.001, **** *p* < 0.0001 caffeine compared to saline, two-way ANOVA with Bonferroni multiple comparisons. Episodes are partitioned into ten exponentially increased duration bins from 10 to >2560 s (x-axis designates the upper limit of each bin for the following bins: 0–10, 11–20, 21–40, 41–80, 81–160, 161–320, 321–640, 641–1280, 1281–2560, >2560). Data are shown as mean ± SEM.

The changes in the amount of waking, NREM sleep, and REM sleep were reflected in the distribution of the vigilance state episodes (Figure 2). Caffeine treatment reduced the number of short (<40 s) NREM sleep episodes during both the rest and active phase on the first day (Figure 2C,D). This change was accompanied by an increase in the number of short (<20 s) waking episodes during the subjective day (Figure 2A). However, there was an opposite effect in the subjective night (the active phase of the animal where it normally is dark) with a reduction in the number of short waking episodes (Figure 2B). Caffeine also changed the duration distribution of the REM sleep episodes, as the ANOVA indicated an overall difference in the number of episodes; however, this did not result in significant results in the post hoc *t*-test for single duration bins (Figure 2E,F). In the rest period immediately after treatment, caffeine clearly increased the number of short waking episodes and reduced the number of short NREM sleep episodes. This, however, seemed to result in the opposite response in the following active period.

On the second recovery day, the reduction in short (<20 s) NREM sleep episodes under both subjective day and night was repeated (Figure 2I,J). During the subjective day, the short (<20 s) waking episodes were decreased on the second recovery day of the caffeine-treated group (Figure 2G). The number of 10 s waking episodes remained lower in the subjective night on the second day after caffeine treatment (Figure 2H). REM sleep episode distribution on the second recovery day remained different after caffeine administration compared to control treatment (Figure 2K,L). Therefore, a prolonged effect of a single dose of caffeine could also be observed here.

### 2.3. Effect of Caffeine on EEG Slow-Wave and Theta Activity

**Figure 3:**
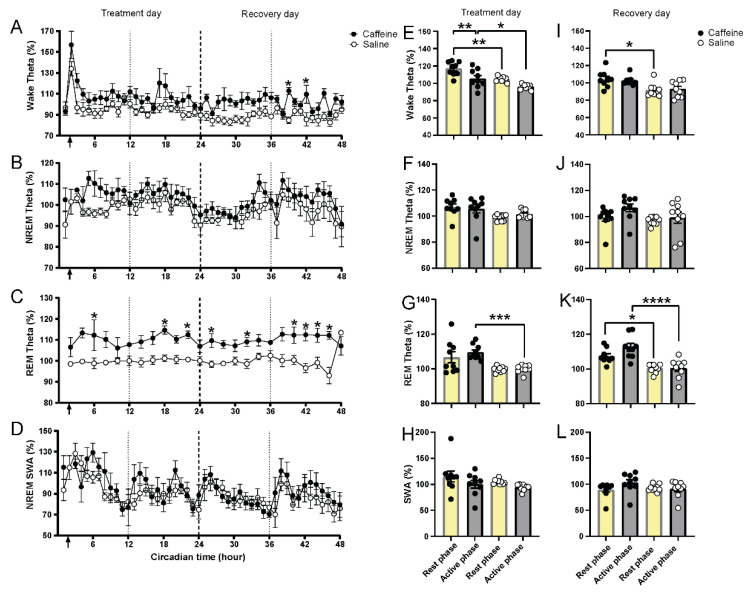
Theta activity in different vigilant states and slow wave activity in NREM sleep for 48 h under constant darkness. (**A**–**D**) Time course of theta activity in waking, NREM, and REM sleep (2 h interval) and slow-wave activity in NREM sleep for caffeine (black, *n* = 9) and saline (white, *n* = 9) administration in 48 h. The first 24 h were considered as the treatment day; the second 24 h were considered as the recovery day. Arrows indicate the injection time (CT1-2). Asterisks indicate significant differences between caffeine and saline administration (* *p* = 0.05–0.0001, Bonferroni multiple comparisons test after significant two-way ANOVA, factors “treatment” or interaction of “circadian time” and “treatment”). (**E**–**H**) Subjective day and night values of theta activity in waking, NREM and REM sleep, and slow-wave activity in NREM sleep for caffeine (black, *n* = 9) and saline (white, *n* = 9) administration on treatment day. (**I**–**L**) Rest and active phase values of theta activity in waking, NREM and REM sleep, and slow-wave activity in NREM sleep for caffeine (black, *n* = 9) and saline (white, *n* = 9) administration on recovery day. CT0-CT12 was considered as the subjective day (yellow bar); CT12-CT24 was considered as the subjective night (gray bar). Asterisks indicate significant differences between caffeine and saline administration or the differences between subjective day and subjective night (* *p* < 0.05, ** *p* < 0.01, *** *p* < 0.001, **** *p* < 0.0001, Bonferroni multiple comparisons test after significant two-way ANOVA). Data are shown as mean ± SEM.

As we observed the long-lasting effect on REM sleep, we decided to analyze theta activity (6.0–9.0 Hz), the most prominent EEG frequency in REM sleep, in all three vigilance states (Figure 3). After caffeine treatment, theta activity was initially increased in all three vigilance states compared to saline (Figure 3A–C). In NREM sleep, values returned to baseline within a couple of hours and no differences were seen over the two days (Figure 3F,J). Increased theta activity in waking persisted for approximately 10 h, resulting in a significant increase in the average value over the rest phase (Figure 3E). On the second recovery day, this increase persisted, resulting in significantly increased theta activity values for both the rest and active phase (Figure 3I). Similarly, but more pronounced, theta activity during REM sleep remained high for the entire 47-h recording period after caffeine administration (Figure 3C,G,K; Table 2). Theta activity in both waking and NREM sleep was dependent on circadian time (Figure 3A,B, Table 2), whereas theta activity in REM sleep did not show a significant circadian rhythm (Table 2).

As slow-wave activity in NREM sleep is a marker of sleep homeostasis and has been shown in the past to be influenced by caffeine [1,4,5,9], we analyzed this variable as well. We found no significant changes in slow-wave activity in response to 15 mg/kg caffeine (Figure 3D,H,L). The slow-wave activity in NREM sleep showed a time-dependent rhythm under constant darkness in Brown Norway rats, as previously shown in other rodents (Figure 3D, Table 2) [35,36]. Maximum activity in the slow-wave range in NREM sleep seemed to be delayed on the first day after caffeine treatment compared to saline. The latter may be due to the increased waking in the first three hours after caffeine treatment.

### 2.4. Effect of Caffeine on the EEG Power Spectrum of Waking and REM Sleep

**Figure 4:**
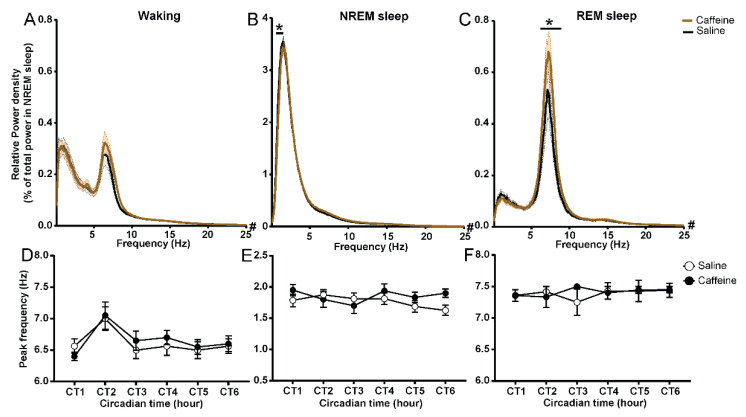
Effect of caffeine on power spectrum of wake, NREM sleep, and REM sleep. (**A**–**C**) Relative EEG power spectrum in waking, NREM sleep, and REM sleep during CT0–CT6 for caffeine (brown, *n* = 9) and saline (black, *n* = 8) administration. # indicates the influence of factor “treatment”. Asterisk indicates significant differences between caffeine and saline administration (* *p* = 0.05–0.0001, Bonferroni multiple comparisons test after significant two-way ANOVA, factors “treatment” or interaction of “frequency” and “treatment”). (**D**–**F**) Peak frequency among 6.0–9.0 Hz in waking and REM sleep, and 0.5–2.5 Hz in NREM sleep during CT0–CT6 for caffeine (black, *n* = 9) and saline (white, *n* = 8) administration. Data are shown as mean ± SEM.

As we observed an overall increase in the theta activity in waking, NREM sleep, and REM sleep, we wanted to understand how caffeine influences the EEG power spectrum compared to saline administration. This is to ensure that the results we found in theta power density were not caused by changes in theta frequency over the course of the experiment. For this purpose, we analyzed the power density spectra (0–25 Hz) of waking and REM sleep after both saline and caffeine treatment, and expressed them relative to the total EEG power density of the NREM sleep EEG obtained on the first day after the saline injection. Caffeine induced an increase in EEG power density of REM sleep specifically in the theta range from 6.6 Hz to 8.1 Hz (Figure 4C), and reduced activity in the slow-wave range (0.6 Hz to 1.5 Hz) in the NREM sleep spectrum (Figure 4B). Caffeine also affected EEG activity in waking (Figure 4A, ANOVA factor “treatment” *p* = 0.0023) relative to saline administration, but no specific frequency bin was distinguished in the post hoc test. To ensure that the theta peak in the waking and REM sleep EEG remained stable after treatment, we determined its frequency for the first 6 h after treatment and found that both for waking and REM sleep, theta peak frequency after caffeine treatment was very similar to the corresponding peak frequency after saline (Figure 4D,F), indicating that caffeine did not shift the peak of theta frequency activity. The theta peak frequency of waking at CT1 was approximately 6.5 Hz, and both caffeine and saline injection increased the peak frequency to 7.0 Hz at CT2. The increased peak frequency after the injection probably reflects the change to more active waking. Additionally, the slow-wave peak in NREM sleep did not shift due to caffeine (Figure 4E). These results indicate that caffeine decreases slow-wave activity during NREM sleep and increases theta power density in REM sleep, but does not change its frequency.

### 2.5. Effect of Caffeine on the Neural Activity in the Lateral Hypothalamus

**Figure 5:**
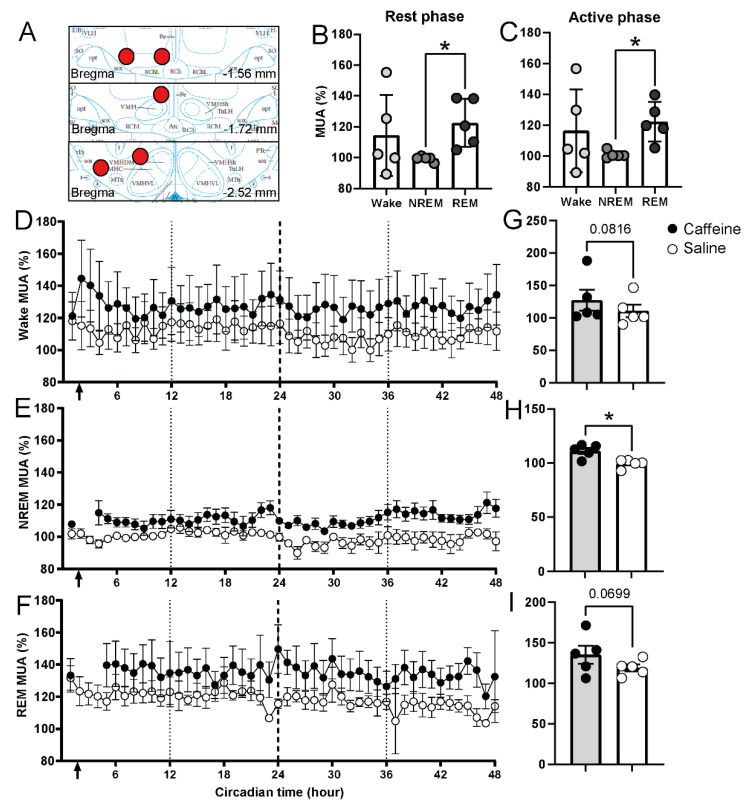
Electrical activity in lateral hypothalamus under caffeine and saline treatment over 48 h. The location of the electrodes in PLH is shown in (**A**). PLH activity under subjective day (**B**) and subjective night (**C**) under baseline condition (saline-treated). CT0-CT12 was considered as the subjective day, CT12-CT24 was considered as the subjective night. Asterisks indicate significant neuronal activity differences between NREM and REM states (* *p* < 0.05, paired *t*-test). (**D**–**F**) Time course of neuronal activity of PLH in waking, NREM, and REM sleep (2 h interval) for caffeine (black, *n* = 5) and saline (white, *n* = 5) administration in 48 h. The first 24 h was considered as treatment day; the second 24 h was considered as the recovery day. Arrow indicates the injection time (CT1-2). (**G**–**I**) Average value of 47 h of neuronal activity of PLH in waking, NREM, and REM sleep for caffeine (black, *n* = 5) and saline (*n* = 5). Asterisk indicates significant differences between caffeine and saline (* *p* = 0.0251, paired *t*-test). Data are shown as mean ± SEM.

Previously, it was found that an overall neuronal activation can be observed immediately after caffeine administration, particularly in the cortex [26]. In the present data, we noticed that there was a long-term rise in the EEG theta power density, which is likely to be associated with increased synchronized activity in the hippocampus [37]. In addition, we observed changes in the amount of REM sleep over two days after treatment. It has been shown previously that 10 and 30 mg/kg caffeine can increase the activity of orexin neurons in LH [27]. This indicates that the LH might be influenced by acute caffeine. We therefore assessed the effect of acute caffeine on LH neuronal activity combined with the sleep–wake changes in our freely moving rats (Figure 5, location of the recording electrode in 5A). In these recordings, PLH electrical discharge rates were significantly higher during REM sleep compared to NREM sleep, both during subjective day and subjective night, but no day–night differences were observed (Figure 5B,C). Waking values were in between NREM and REM sleep discharge rates. After caffeine administration, PLH neural activity was increased in all three vigilance states (Figure 5D–F, Table 3). As no day–night fluctuations were observed, the neuronal activity after injection under waking, NREM, and REM sleep was averaged over the 47 h remaining for the recording. The neuronal activity during NREM sleep was significantly higher after caffeine administration (Figure 5H). A similar increase in neuronal activity during waking and REM sleep only showed a trend (Figure 5G,I). This indicates that the changes after a single application of caffeine in PLH neuronal activity also last for two days.

## 3. Discussion

Caffeine is one of the most widely used stimulators, significantly promoting wakefulness. In the past decades, several researchers have documented the effects of caffeine on sleep and the circadian clock [6,38,39,40]. However, most of these studies were either short-term or did not exclude the influence of the light–dark cycle. In the present study, we tested the effects of caffeine, given 1 h after the onset of the endogenous rest phase, on vigilance states, the EEG, and neuronal activity in PLH over two days under constant dark conditions. Caffeine reduced NREM and REM sleep in the initial 3 h after the injection compared with saline, which is in accordance with previous findings in albino rats [1]. However, in addition, we found that caffeine influenced the occurrence of REM sleep for almost 36 h and REM sleep theta activity in the EEG for the whole recording duration (47 h). Correspondingly, neuronal activity in the PLH was increased during this same period. These findings confirm our idea that the effect of a single dose of caffeine may be longer than previously envisioned. We recorded the data in constant dark conditions, whereas previous experiments were all performed in a light–dark cycle. This may be why we were able to observe these long-term changes, and suggests that the influence of caffeine on sleep and circadian rhythms lasts longer in the absence of a light–dark cycle, or when light levels are low.

### 3.1. Acute Effect of Caffeine on Vigilance States

There are several candidate brain areas that are putative targets of caffeine to induce waking and activity. The administration of 10 mg/kg caffeine in rats leads to an acute and widespread increase in the rate of cerebral glucose utilization in the nucleus accumbens, both the shell and the core, as well as in most structures of the extrapyramidal motor system, and in many limbic regions and cortices, which may decrease sleep. Furthermore, the basal forebrain and mesopontine tegmentum, which are arousal-promoting areas, are activated following systemic caffeine injection [41]. Caffeine may also inhibit the activation of sleep-promoting brain areas such as the ventrolateral preoptic nucleus and block the sleep-promoting adenosine receptor-mediated effects of adenosine [42,43].

In most previous studies, the effect of caffeine was examined under the influence of a light–dark cycle. As light is a strong zeitgeber for the sleep–wake cycle, the effect of caffeine on sleep will be influenced by light, or an interaction between caffeine and light may occur in these studies. Caffeine is known to significantly affect the amplitude of peripheral clocks [44]. To illuminate the effect of light, we kept our rats in constant dark conditions and administered caffeine at CT1. In our 48 h recording, caffeine treatment initially reduced sleep and increased locomotor activity, which is in line with previous sleep studies in rodents [1,45,46,47]. It is well known that the enhanced wakefulness and activity after caffeine administration is mainly caused by a blockage of adenosine receptors in the brain [6,48].

However, in addition, we observed that the amount of REM sleep was still reduced on the second day after caffeine administration. The pharmacokinetics of 15 mg/kg caffeine in rats is relatively well known. Plasma levels of caffeine are known to steadily decline, but caffeine is still elevated 8 h after application [1]. It is unlikely that levels are still high on the second day, 24 h after administration. However, on the second day after application, the amount and the circadian modulation of REM sleep were still reduced in our study. As REM sleep is under strong influence of the circadian clock [49], this may be due to a disturbance of the circadian clock by caffeine on the previous day or due to an influence of caffeine metabolites on the second day. Caffeine is known to phase delay, or slow down the circadian clock [21,22,24,40]. The circadian clock is synchronized to the normal 24 h rhythm by the external light–dark cycle. As the light–dark cycle was absent in our experiment, the effect of caffeine may be increased in our study, which may have helped to reveal the prolonged influence of caffeine or caffeine metabolites on REM sleep. These data suggest that a single dose of caffeine during the rest phase increases the voluntary movement and influences sleep architecture during the first half day and that minor but significant changes in sleep can still be present on the second day, particularly if light is absent or light levels are kept low.

### 3.2. Theta Activity in Different Sleep Stages

Another remarkable finding was that in the EEG of all the vigilance states, we found a specific increase in theta activity. This lasted for approximately 11–12 h in waking and NREM sleep and for more than 47 h in REM sleep. We ensured that the peak frequency did not become faster or slower after caffeine treatment and established that the increase was a genuine increase in power density in the same frequency range as the peak frequency in the control condition.

In rodents, it is well known that the theta waves in the cortical EEG during REM sleep and waking are mainly generated in the hippocampus [37]. These 6–9 Hz frequency waves have been associated with cognitive and memory performance, and voluntary movement [50,51]. Theta activity during waking was shown to be increased during sleep deprivation, which is probably due to increased activity and movement [52]. Reportedly, EEG theta oscillations during waking are an electrophysiological sign of locomotor activity and voluntary behavior in mice [53]. Due to the ability of caffeine to block the action of adenosine, caffeine has a stimulatory action on norepinephrine, dopamine, acetylcholine, serotonin, glutamate, and gamma-aminobutyric acid neurons, which can activate the motor circuit in the brain [54].

In our recordings, we found a consistent increase in theta activity in the two days after treatment. This was particularly strong in REM sleep. Both the reduction in circadian amplitude of REM sleep and the increased theta on the second day may indicate that caffeine, or its metabolites, have a particularly strong influence on REM sleep variables.

The peak frequency of the theta activity is slower in waking (~6.5 Hz) compared to REM sleep (~7.5 Hz) in our animal model, the Brown Norway rat. A recent study showed that increased theta activity in REM sleep was associated with high energy cost [55]. Caffeine, and possibly its metabolites, can regulate the metabolic rate and energy balance. Some studies show that caffeine can decrease body weight and increase energy expenditure [56,57]. This may explain the higher theta activity during REM sleep over 2 days; however, the underlying mechanism is not clear.

### 3.3. Activity in of the Lateral Hypothalamus

We recorded neuronal activity in different vigilance states in the PLH because previous research showed that orexin neurons in this area show increased c-fos expression after caffeine treatment [27]. We show that the activity differs between the different states, with high activity during REM sleep, intermediate activity during waking, and the lowest activity during NREM sleep. Remarkably, we did not observe a difference in activity between day and night, although in the past, it was shown that orexin (hypocretin) levels, which are dependent on neurons in the LH, show a circadian modulation [58,59]. In parallel with the increased theta in the cortex, we observed an increased neuronal firing rate in the PLH after caffeine treatment. This increase was most pronounced and significant during NREM sleep.

The hypothalamus is the central regulator of energy homeostasis in animals [60]. Caffeine administered at 10, 30, and 75 mg/kg in rats significantly increased c-fos immunoreactivity in dorsomedial and LH orexin neurons, which are thought to play an important role in arousal state control and energy expenditure [27,61,62]. An earlier study reported that caffeine promotes glutamate and histamine in the posterior hypothalamus (PH) [63]. A lower dose of caffeine may further activate the histaminergic neurons. Histamine maintains wakefulness through direct projections of PH histamine neurons to the thalamus and the cortex [63]. This suggests that the neural pathways in the hypothalamus are activated by caffeine, which may induce an increase in neural activity in the cortex, causing increases in locomotor activity, alertness, and energy expenditure. In this context, it is interesting that hypocretin receptors are also found in the hippocampus [64]. As the theta waves observed in the EEG are mainly generated in the hippocampus [37], this suggests that our finding of simultaneous increased activity in the PLH and increased theta activity in the cortical EEG may be connected to a common source.

## 4. Materials and Methods

### 4.1. Animals

Thirteen 12-week-old male Brown Norway rats (Charles River) were used in this study. Rats were group-housed under 12:12 light–dark conditions (lights on 8:00, lights off 20:00) with food and water ad libitum in a temperature-controlled room (21–22 °C). All animal experiments were approved by the Central Committee on Animals Research (CCD, The Netherlands) and were carried out in accordance with the EU Directive 2010/63/EU on the protection of animals used for scientific purposes.

### 4.2. Surgery

At a body weight of approximately 250 g (12 weeks of age), the animals were put under deep anesthesia with ketamine (Aescoket, Boxtel, The Netherlands; 65 mg/kg) and xylazine (Rompun, Bayer AG, Leverkusen, Germany; 13.3 mg/kg). The in vivo neuronal activity and EEG/EMG surgery techniques were used as described previously [35]. Two electrodes were aimed at the LH while the other electrode, with the insulation completely removed, was placed in the cortex for reference. For EEG, electrodes (Plastics One, Roanoke, VA, USA) were screwed through the skull on the dura over the right cortex (2.0 mm lateral to the midline, 3.5 mm posterior to bregma) and the cerebellum (at the midline, 1.5 mm posterior to lambda). Two wires with suture patches (Plastics One) were inserted between the skin and the neck muscle tissue for EMG recordings. The wire branches of all electrodes were set on a plastic pedestal (Plastics One, Roanoke, VA, USA), which was fixed to the skull with dental cement and three additional support screws. The rats were allowed to recovered for at least seven days following the surgery under 12:12 LD conditions. After fully recovering from the surgery for at least seven days, the animals were connected to the recording system by a flexible cable and a counterbalanced swivel system, and they remained on the cable under constant darkness for at least one week before the start of the recording. The recording chamber was equipped with a passive infrared sensor and drinking sensor [35,36]. The animals’ locomotor activity and drinking activity were recorded continuously to obtain an estimate of the circadian phase. From this, onset and offset of rest and activity were determined, and an F-periodogram analysis provided an estimate of circadian period. This enabled us to determine the time of treatment for the next recording day. All animals were free running, but with a minimal deviation from 24 h (mean 24.2 ± 0.04, range: 24.2–24.8, *n* = 13), which meant that we did not correct circadian phase over the two-day recording period.

### 4.3. Drug Treatment

Caffeine (Merck, C0750, Rahway, NJ, USA) was dissolved in 0.9% saline (LUMC Pharmacy, Leiden, The Netherlands) at a concentration of 15 mg/mL. The concentration was used previously in several studies, and is known to significantly increase waking in rats and mice [1,65], as caffeine metabolism differs between rodents and humans; this dosage corresponds to ~5 mg/kg in humans, which is about 3 to 4 cups of coffee (each containing ~100 mg caffeine) [6]. Under constant darkness, the animals received either a caffeine (15 mg/kg) or saline (1 mL/kg) intraperitoneal injection under a randomized cross-over design at circadian time 1 (CT1). CT1 was determined by the clear on- and offset of locomotor activity (CT0) on the days before the injection. At least 3 circadian days were given between two treatments. On average, the time between treatments was 6 days (±0.6) with a range of 3–10 days.

### 4.4. EEG Data Acquisition

The EEG and EMG were simultaneously and continuously recorded for 48 h, as previously described [35]. The signals were amplified (amplification factor ~2000), bandpass-filtered (EEG: 0.5–30.0 Hz, −40 dB/decade; EMG 15.0–40.0 Hz, −40 dB/decade), digitized (sampling rate 100 Hz) in 10 s epochs, and automatically stored on a hard disk (Spike2, Power1401, CED, Cambridge, UK). A fast Fourier transformation routine with a 10 s window was performed offline (MATLAB, The MathWorks Inc., Natick, MA, USA) to compute EEG power density spectra within the frequency range 0.1–25.0 Hz. PLH/LA neuronal activity was recorded online (amplification factor ~50,000, bandpass-filtered between 500 and 5000 Hz, −40 dB/decade). A window discriminator converted the recorded action potentials to electronic pulses. A second window discriminator was set at a higher level to be able to exclude artifacts caused by the animal’s movements. The EEG and EMG were continuously recorded and amplified (amplification factor ~2000), bandpass-filtered (between 0.5 and 30 Hz, −40 dB/decade), and subjected to analog-to-digital conversion (sampling rate 100 Hz). All data were recorded simultaneously in 10 s epochs.

### 4.5. Data Analysis

Three vigilance states (waking, NREM, and REM sleep) were scored offline in 10-s epochs. The manual scoring of vigilance states based on the EEG and EMG recordings was performed according to standardized criteria for rats [35,66]. Waking, NREM, and REM sleep were determined, and artifacts were excluded for power spectral analysis. In three animals, the recording after the control injection did not last the full two days. Therefore, we had 10 animals in the control condition for the calculation of the vigilance states. Due to problems with the quality of the recordings, one animal did not contribute to the EEG spectral analysis data. Since for the spectral analysis, it was necessary to re-calculate power density values relative to the first day of the control condition, complete and clean recordings were needed from all animals for both conditions to enter the analysis. Unfortunately, this was not possible in one animal, which was therefore excluded from the analysis of the EEG power density spectra (*n* = 9 left). To investigate the effect of caffeine on EEG power density of different vigilance states, we analyzed the EEG power density in the slow-wave range (SWA, 1.0–4.0 Hz) in NREM sleep, and theta range (6.0–9.0 Hz) in waking, NREM, and REM sleep, as described previously [35]. The average amount of the vigilance states (waking, NREM sleep, REM sleep, and REM sleep per total sleep time) and EEG spectral data (NREM-SWA, theta) were analyzed in 1 h intervals and 12 h intervals over 48 h in both caffeine and saline treatment. The locomotor activity was collected in 10 s intervals, and was further averaged in 1 h and 12 h intervals. Spectral analysis was performed using fast Fourier transform (FFT; 0.1–25 Hz, 0.1 Hz resolution); the relative power density spectra of waking and REM sleep in caffeine and saline day (CT0-CT6) were analyzed. The peak frequency of waking (theta range) and REM sleep was determined in hourly values in the same 6 h. Sleep–wake state episodes were determined with an algorithm described previously [67]. Episodes of each vigilance state were partitioned into a maximum of ten bins with the exponentially increased duration from 10 s to >2560 s. All PLH neuronal activity and EEG power density data were standardized relative to the mean 24 h saline control injection day in NREM sleep. This enabled the calculation of mean values for the rats which had histological verification of the electrode recording site in the PLH area (*n* = 5).

### 4.6. Histology

After the recording, the mice were sacrificed in a CO_2_ chamber, and a small electrolytic current (1.2 mA, 10 s) was passed through the two twisted electrodes to mark their positions. The brains were collected and kept in a 4% paraformaldehyde solution containing ferrocyanide to fix the brain tissue and to stain the recording site. After fixation, the brain was transferred into 30% sucrose and sectioned coronally and stained with cresyl violet. The marked position of the electrode was verified by microscopic inspection.

### 4.7. Statistics

For data analysis, Prism 8 software (Graphpad Inc., La Jolla, CA, USA). was used. Two-way ANOVA with Bonferroni multiple comparisons was used to compare the effects of treatment across time, the effects of treatment across different EEG power frequencies, and the effects of treatment across different episode durations. Detailed statistics of two-way ANOVA are shown in Table 1, Table 2 and Table 3. Paired *t*-tests were used for circadian 12 h intervals (rest or active phase) of multi-unit activity under different vigilance states. Significant differences between conditions are represented in the graphs as follow: * *p* < 0.05; ** *p* < 0.01; *** *p* < 0.001; **** *p* < 0.0001.

## 5. Conclusions

In summary, we found that a single dose of caffeine not only increases waking and decreases sleep in the short term (first 3 h after administration), but also influences REM sleep and theta activity in REM sleep on the following day. In addition, neuronal activity in PLH was increased for approximately two days, particularly in NREM sleep. One of the limitations of our studies is that we only performed these recordings in male rats and therefore cannot determine whether similar changes occur in females. A limitation of the recordings of neuronal activity in the PLH is that we only recorded from a small number of animals (*n* = 5), and that we were unable to determine the type of neurons from which we recorded spiking activity. Our recordings were performed in constant darkness, which may have increased the opportunity to observe these long-term effects, as the light–dark cycle may reduce the influence of the circadian clock and reduce the effect of caffeine. The latter could also be of importance in humans. Considering the interactive effects of light and caffeine on the sleep and circadian rhythms, it may be important to consider lighting levels during the day when investigating the effects of caffeine in humans.

## Figures and Tables

**Table 1:** Detailed statistics for two-way ANOVA of 48 h vigilance stages and PIR data.

Vigilance State and PIR	Time of Day	Treatment	Interaction
**Treatment day**			
Waking	F (23, 480) = 3.159	F (1, 480) = 4.427	F (23, 480) = 2.565
	*p* < 0.0001	*p* = 0.0359	*p* = 0.0001
NREM sleep	F (23, 480) = 2.718	F (1, 480) = 3.564	F (23, 480) = 2.727
	*P* = 0.0596	*p* = 0.0596	*p* < 0.0001
REM sleep	F (23, 480) = 3.645	F (1, 480) = 4.995	F (23, 480) = 1.474
	*p* < 0.0001	*p* = 0.0259	*p* = 0.0733
REM/Total sleep	F (23, 451) = 2.050	F (1, 451) = 4.157	F (23, 451) = 0.8427
	*p* = 0.0031	*p* = 0.0420	ns ^1^
PIR	F (23, 480) = 9.019	F (1, 480) = 6.516	F (23, 480) = 4.506
	*p* < 0.0001	*p* = 0.0110	*p* < 0.0001
**Recovery day**			
Waking	F (23, 456) = 2.291	F (1, 456) = 0.2185	F (23, 456) = 0.6089
	*p* = 0.0007	ns	ns
NREM sleep	F (23, 456) = 2.361	F (1, 456) = 0.08843	F (23, 456) = 0.5142
	*p* = 0.0004	ns	ns
REM sleep	F (23, 456) = 2.964	F (1, 456) = 4.994	F (23, 456) = 1.052
	*p* < 0.0001	*p* = 0.0259	ns
REM/Total sleep	F (23, 442) = 1.979	F (1, 442) = 0.6342	F (23, 442) = 1.025
	*p* = 0.0048	ns	ns
PIR	F (23, 480) = 4.663	F (1, 480) = 0.5301	F (23, 480) = 0.6357
	*p* < 0.0001	ns	ns

^1^ ns: not significant.

**Table 2:** Detailed statistics for two-way ANOVA of 48 h theta activity and SWA.

States	Time of Day	Treatment	Interaction
**Treatment day**			
Theta in waking	F (23, 384) = 5.844	F (1, 384) = 45.62	F (23, 384) = 0.8229
	*p* < 0.0001	*p* < 0.0001	ns ^1^
Theta in NREM sleep	F (23, 345) = 1.676	F (1, 345) = 25.89	F (23, 345) = 0.5991
	*p* = 0.0278	*p* < 0.0001	ns
Theta in REM sleep	F (11, 153) = 0.5196	F (1, 153) = 60.51	F (11, 153) = 0.3431
	ns	*p* < 0.0001	ns
SWA in NREM sleep	F (1, 339) = 5.955	F (22, 339) = 3.652	F (22, 339) = 0.8643
	*p* < 0.0001	*p* = 0.0152	ns
**Recovery day**			
Theta in waking	F (23, 384) = 1.174	F (1, 384) = 80.43	F (23, 384) = 0.9871
	ns	*p* < 0.0001	ns
Theta in NREM sleep	F (23, 362) = 2.214	F (1, 362) = 15.37	F (23, 362) = 0.3396
	*p* = 0.0012	*p* = 0.0001	ns
Theta in REM sleep	F (11, 144) = 0.5267	F (1, 144) = 59.84	F (11, 144) = 1.162
	ns	*p* < 0.0001	ns
SWA in NREM sleep	F (23, 362) = 2.491	F (1, 362) = 1.901	F (23, 362) = 0.5437
	*p* = 0.0002	ns	ns

^1^ ns: not significant.

**Table 3:** Detailed statistics for two-way ANOVA of neuronal activity under different sleep stages.

States	Time of Day	Treatment	Interaction
Treatment day			
Waking	F (23, 192) = 0.1205	F (1, 192) = 10.85	F (23, 192) = 0.1161
	ns ^1^	*p* = 0.0012	ns
NREM sleep	F (21, 173) = 0.6057	F (1, 173) = 74.28	F (21, 173) = 0.5898
	ns	*p* < 0.0001	ns
REM sleep	F (20, 146) = 0.1174	F (1, 146) = 19.31	F (20, 146) = 0.2023
	ns	*p* < 0.0001	ns
Recovery day			
Waking	F (23, 192) = 0.1227	F (1, 192) = 21.47	F (23, 192) = 0.06239
	ns	*p* < 0.0001	ns
NREM sleep	F (23, 188) = 1.282	F (1, 188) = 144.3	F (23, 188) = 0.3960
	ns	*p* < 0.0001	ns
REM sleep	F (23, 158) = 0.3248	F (1, 158) = 46.23	F (23, 158) = 0.1461
	ns	*p* < 0.0001	ns

^1^ ns: not significant.

## Data Availability

The data presented in this study are available on request from the corresponding author. The data are not publicly available due to incompatibility with existing repositories.
